# Stem Cells to the Rescue: Development and Application of Cell-Based Therapy for Microvascular Repair

**DOI:** 10.3390/cells10082144

**Published:** 2021-08-20

**Authors:** Lilach O. Lerman, Amir Lerman

**Affiliations:** 1 Division of Nephrology and Hypertension, Mayo Clinic, 200 First Street SW, Rochester, MN 55905, USA; 2 Department of Cardiovascular Medicine, Mayo Clinic, 200 First Street SW, Rochester, MN 55905, USA

The microcirculation includes an invisible network of micro-vessels that are up to a few hundred microns in diameter. The microcirculation in each organ is largely responsible for end organ perfusion and the transfer of oxygen from red blood cells to the cells of the parenchyma to meet their energy requirements. In addition, the microcirculation drives a dynamic and demand-driven exchange of solutes between the intra-vascular and extra-vascular spaces, as well as the delivery of nutrients and blood-born humoral substances to tissues. Moreover, the microcirculation may exert these effects via the regulation of vascular tone.

In many tissues, the structural or functional loss of micro-vessels characterizes disease progression and correlates with ultimate organ failure [1]. Damage to the microcirculation can be caused by ischemia, inflammation, or hypoxia [2], which may induce endothelial activation, with the adhesion and extravasation of leukocytes into tissues. Microvascular integrity is maintained by a careful balance between endothelium-derived vasodilators such as nitric-oxide and vasoconstrictors such as endothelin. Exposure to cardiovascular risk, genetic, and epigenetic factors can cause dysfunction or physical injury to the endothelial layer, such as the loss of its integrity, and therefore the impairment of myriad functions attributed to the microcirculation [3], including insulin sensitivity [4]. In addition, the loss of angiogenic signaling, development of tissue fibrosis, prolonged periods of vasoconstriction, and inflammation can cause microvascular obliteration.

One of the mechanisms by which the microcirculation retains its integrity involves a continuous process of endothelial cell repair and replacement, as well as ongoing stimuli to facilitate and foster its expansion. The endogenous cellular repair system plays an important role in the maintenance of the endothelium, as well as parenchymal cells. This system includes bone marrow-derived blood-borne endothelial progenitor cells that continuously patrol the microcirculation to repair and replace injured endothelial cells and micro-vessels whenever and wherever encountered [5]. Upon injury, signals are released from the injured organ to increase the number of endothelial progenitor cells in the peripheral circulation and the cells home to injury sites [6]. In addition to circulating cells, many tissues contain niches of resident stem cells, such as mesenchymal stem/stromal cells, that respond to local cues and are recruited upon injury to repair tissue.

However, these potent reparative systems can be injured or lost during development of systemic or local tissue disease, which may render them ineffective when a tissue requires reparative activities [7]. This apparent structural and functional loss of the microcirculation, together with the inability of the endogenous repair system to restore it, has generated the impetus to exogenously replenish this system. Endothelial progenitor cells seem to have a particularly robust pro-angiogenic activity [8]. Nevertheless, MSCs show potent anti-inflammatory and immunomodulatory activities, and they can both directly and indirectly restore the microcirculation and improve its function [9]. Therefore, both these interventions have been used and gained popularity for the purpose of microvascular repair.

Being a fundamental pathogenic mechanism underlying tissue damage, microvascular repair has been positioned at the center of a number of therapeutic interventions. This Special Issue of *Cells* focuses on the development and application of cell-based therapy for microvascular repair. The ubiquitous nature of microvascular damage as a fundamental mechanism of tissue injury is reflected in the variety of organs in which cell-based therapy has been attempted. The disease entities described in this Special Issue include renovascular disease associated with ischemic nephropathy in both humans [10] and pigs [11], lung injury induced with Influenza A virus in mice [12], coronary microvascular repair [13], and ischemic stroke [14,15]. Novel approaches to achieve microvascular repair via the co-transplantation of kidney tissue-forming cells with vessel-forming cells, as well as sophisticated imaging techniques that are capable of illustrating the morphology and function of the renal microcirculation [16], are described [17]. Importantly, a review article also provides some cautionary notes and describes the reality of clinical development and the limitations that it imposes on the field [18].

Clearly, additional development is needed for the field of cell-based and cell-derived therapy, which is rapidly moving forward. For example, the use of stem cell-derived extracellular vesicles may alleviate some safety concerns associated with the delivery of live replicating cells [19], and the delivery of organoids might allow for the replacement of basic functional units of different tissues in forms that might expedite the restoration of organ function [20]. Novel scaffolds are being designed to ensure long-term residence of the stem-cells in tissues and increase their ability to release favorable paracrine mediators and endow healing benefits [21]. The genetic engineering of stem cells [22] or their daughter extracellular vesicles [23] may improve tissue targeting and enable the utilization of these cells and cellular products as vehicles to deliver additional drugs or diagnostic features [24].

Overall, teamwork, scientific collaboration, and knowledge dissemination, as exemplified in the current Special Issue of *Cells*, will hopefully propel this field forward in the coming years.
